# Variation in Metal–Support Interaction with TiO_2_ Loading and Synthesis Conditions for Pt-Ti/SBA-15 Active Catalysts in Methane Combustion

**DOI:** 10.3390/nano13101647

**Published:** 2023-05-15

**Authors:** Mihaela Filip, Elena Maria Anghel, Vasile Rednic, Florica Papa, Simona Somacescu, Cornel Munteanu, Nicolae Aldea, Jing Zhang, Viorica Parvulescu

**Affiliations:** 1 Ilie Murgulescu Institute of Physical Chemistry, Romanian Academy, Splaiul Independentei 202, 060021 Bucharest, Romania; mihaela_burcin@yahoo.com (M.F.); floricapapa@gmail.com (F.P.); ssimona@icf.ro (S.S.);; 2 National Institute for R&D of Isotopic and Molecular Technologies, Donat St. 67-103, 400293 Cluj-Napoca, Romania; 3 Beijing Synchrotron Radiation Facilities of Beijing Electron Positron Collider National Laboratory,19B Yuquan Road, Beijing 100049, China

**Keywords:** mesoporous catalysts, Pt/Ti-SBA-15, peroxotitanate, tetrabutylorthotitanate, titania loading, metal-support interaction, CH_4_ oxidation

## Abstract

The control of catalytic performance using synthesis conditions is one of the main goals of catalytic research. Two series of Pt-Ti/SBA-15 catalysts with different TiO_2_ percentages (*n* = 1, 5, 10, 30 wt.%) were obtained from tetrabutylorthotitanate (TBOT) and peroxotitanate (PT), as titania precursors and Pt impregnation. The obtained catalysts were characterized using X-ray diffraction, scanning electron microscopy (SEM) and transmission electron microscopy (TEM), N_2_ sorption, Raman, X-ray photoelectron spectroscopy (XPS), X-ray absorption spectroscopy (XAS), hydrogen temperature-programmed reduction (H_2_-TPR) and H_2_-chemisorption measurements. Raman spectroscopy showed framework titanium species in low TiO_2_ loading samples. The anatase phase was evidenced for samples with higher titania loading, obtained from TBOT, and a mixture of rutile and anatase for those synthesized by PT. The rutile phase prevails in rich TiO_2_ catalysts obtained from PT. Variable concentrations of Pt^0^ as a result of the stronger interaction of PtO with anatase and the weaker interaction with rutile were depicted using XPS. TiO_2_ loading and precursors influenced the concentration of Pt species, while the effect on Pt nanoparticles’ size and uniform distribution on support was insignificant. The Pt/PtO ratio and their concentration on the surface were the result of strong metal–support interaction, and this influenced catalytic performance in the complete oxidation of methane at a low temperature. The highest conversion was obtained for sample prepared from PT with 30% TiO_2_.

## 1. Introduction

Titania has attracted much attention as a catalyst and/or catalyst support, with applications in catalysis and photocatalysis [1,2,3,4]. The increased interest in TiO_2_ supports is due to the strong metal–support interaction (SMSI) existing between noble metals and this oxide [5,6,7,8] having an important role in heterogeneous catalysis [6,7,8,9,10]. Excellent performances were obtained for platinum in catalytic reactions, such as complete or selective oxidations carried out in the gas [11,12,13,14] or liquid phase [15,16]. The catalytic performances of Pt, as well as other noble metals, are intimately related to particle size, which is a crucial parameter influencing the activity, selectivity and lifetime of a catalyst [6,17,18]. For this reason, the most commonly used strategy in the preparation of catalysts with Pt is to deposit platinum nanoparticles on large surface area supports, with the aim of improving the dispersion [19,20]. Unfortunately, titania supports have the disadvantage of a small surface area. Therefore, novel materials were obtained through the immobilization of Ti species into the framework or onto the surface of mesoporous silica, such as MCM-4 [20], HMS [21] SBA-15 [6,18,21,22,23,24], and KIT-6 [25]. Hence, the obtained mesoporous materials with large specific surface area and pore volume exhibit typical properties due to the synergistic activity of TiO_2_ and mesoporous silica [18,26].

Well-ordered mesoporous SBA-15 silica, with a 2D hexagonal arrangement of pores, high surface area, and stability, has been highlighted as an ideal support. The immobilization of metal oxides and metal nanoparticles onto SBA-15 silica favors their dispersion [27,28]. Thus, a stronger metal–support interaction (SMSI) was evidenced for Pt/xTiO_2_-SBA-15 nanocomposites than for classical Pt/TiO_2_ P25 catalysts [29,30]. The use of mesoporous silica-ordered channels as hosts for titanium species is a way to control and stabilize small-size particles and thus adjust interaction with Pt through the selection of the synthesis method, composition, and precursors. The most commonly used methods for titania insertion into a high surface area silica matrix are direct synthesis and impregnation in a post-synthesis method [22,25,29]. Further interaction between the inserted Ti species and the active Pt species triggers the change in the Pt’s existence status and dispersion [20]. Metal–support interaction was originally explained as an electron transfer or the formation of intermediate phases [31,32]. SMSI appears at the metal–TiO_2_ interface, where the oxide is reducible and is favored by dispersion on high surface area silica. It was thus shown that the crystal size of titania is an important parameter allowing us to control SMSI [29]. The effect of TiO_2_ content on Pt–support interaction and catalytic properties in methane combustion was correlated with the homogeneous dispersion of platinum and supported oxides, such as titania and ceria, in KIT-6 silica or SBA-15 [24,25]. However, the role of the titanium precursor and synthesis conditions on platinum interaction with titanium species immobilized on mesoporous silica is not fully known. Tuning the SMSI effect for the optimum catalytic performance of supported Pt using the synthesis method and titania loading is a less commonly approached direction in the complete oxidation of CH_4_ [33,34,35].

Although Pt shows lower catalytic activity in methane oxidation compared to Pd, it is more stable and resistant to sulfur poisoning [36,37]. A redox mechanism for total methane oxidation assumes an optimal metal/oxide ratio of active centers to be available in the case of catalysts based on noble metals [38]. This is a consequence of the thermodynamic equilibrium between dispersed oxide clusters and metallic surfaces. Methane conversion and sulfur tolerance were also improved by the incorporation of an optimum TiO_2_ mass percent in high surface area silica, with specific characteristics of acidity and morphology [21]. The promoter effect of TiO_2_ consists of a strong interaction with a noble metal, which influences the equilibrium between the metal and metal oxide phases. Given the fact that the sensitivity of methane oxidation to the noble metal dispersion on the silica matrix and its interaction with support has been the subject of many studies [7,25,27,31,32,39], complete oxidation of methane can be used as a test reaction in the current work.

The aim of this paper is to prepare various PtTi-SBA-15 mesoporous catalysts and to study the influence of a titanium precursor and Pt/TiO_2_ mass percent ratio on the SMSI effect in catalytic performances during the complete oxidation of CH_4_. Two series of Pt-based catalysts supported on Ti-modified SBA-15 will be prepared using two-step synthesis, in which the titanium precursor is added during the preparation of the mesoporous silica from two different precursors and Pt is added by impregnation.

## 2. Materials and Methods

### 2.1. Materials

Tetraethyl orthosilicate (TEOS), HCl (37%), triblock copolymer P123, hexachloroplatinic (IV) acid hexahydrated (H_2_PtCl_6_·6H_2_O) (from Sigma Aldrich, St. Louis, MO, USA) tetra butyl ortho-titanate (TBOT, 99 wt%, ACROS Organics, Waltham, MA, USA), and hydrogen peroxide (H_2_O_2_-30%, SC. Silal Trading, Bucharest, Romania) were employed as starting materials. A CH_4_ gas mixture (10% CH_4_, 5% N_2_, 85% Ar) from Linde Gas (Linde Gas, Bucharest, Romania) was used for catalytic tests.

### 2.2. Catalysts Preparation

A clear orange-colored solution of peroxotitanate was obtained, according to the procedure proposed by Sánchez et al. [40], from TBOT and H_2_O_2_ solutions in water.

The PtTi-SBA-15 mesoporous catalysts were prepared in two steps. In the first step, two series of Ti-SBA-15 supports with different TiO_2_ mass percents were synthesized. The first series of supports, with 1, 5, 10, and 30% titania mass percentage, was obtained from TBOT as a titanium precursor, and the second, with 5, 10, and 30% TiO_2_, from the obtained peroxotitanate (PT). For both syntheses, 4 g of triblock copolymer P123 was dispersed in 30 mL of bidistilled water under continuous stirring at 40 °C. After 30 min an acidic solution, a slurry was obtained by adding 110 mL of aqueous solution of HCl (2M). The slurry was continuously stirred for 2 h before a mixture of TEOS and titanium precursor was added. The obtained gels were transferred into Teflon-lined autoclaves and hydrothermally treated for 48 h at 100 °C. The solid fraction was filtered, rinsed with bidistilled water, and dried for 6 h at room temperature and overnight at 80 °C. Finally, the samples were calcined at 550 °C in the air (temperature ramp of 2 °C min^−1^).

The obtained supports were impregnated, in the second step, with an aqueous solution of H_2_PtCl_6_. After impregnation, the samples were dried overnight at 80 °C and cooled down at room temperature. The final nanocomposite samples with 0.25% wt. Pt calcined in air at 400 °C, were named PTnSB (samples obtained with TBOT), and PTnSP in the case of PT titania precursor (*n* stands for 1, 5, 10, and 30% wt. TiO_2_ loading).

### 2.3. Catalysts Characterization

The structural properties of Ti-SBA-15 supports and PtTi-SBA-15 catalysts were characterized using powder X-ray diffraction by means of a Rigaku Ultima IV diffractometer (Rigaku Corporation, Tokyo, Japan) with Cu Kα (λ = 0.15406 nm). The textural characteristics were determined using a Micromeritics ASAP 2020 instrument (Norcross, GA, USA). The morphology, sample composition, homogeneous dispersion of components, and ordered porous structure were examined using a scanning electron microscope (SEM with EDX, FEI Quanta 3D FEG scanning microscope from FEI, Brno, Czech Republic) and a transmission electron microscope (TEM, Tecnai 10 G2-F30 from Thermo Fisher Scientific, Waltham, MA, USA).

Micro-Raman spectra of the Pt-containing materials were collected using a LabRam HR800 spectrometer (Horiba France SAS, Palaiseau, France) calibrated with a silicon wafer as a reference. Samples were excited with two laser lines (532 nm and 325 nm) through x50LWD/0.55 NA and x40/0.47NUV air objectives, respectively, of an Olympus microscope (Olympus Corporation, Tokyo, Japan). The Raman signal was energy-dispersed by the 600-line/mm and 2400 diffraction gratings. The spectral resolution was better than 2 cm^−1^.

XPS experiments were carried out on a photoelectron spectrometer (XPS-PHI Quantera equipment, Ontario, Canada). The X-ray source was Al Ka radiation (1486.6 eV, monochromatized), and the total overall energy resolution was estimated at 0.65 eV by the full width at half maximum (FWHM) of Au4f_7/2_ line. To consider the charging effect on the measured binding energies (BEs), the spectra were calibrated using the C1s line (BE = 284.8 eV, C-C (CH)_n_ bonding) of the adsorbed hydrocarbon on the sample surface. A dual beam-neutralizing procedure (e^−^ and Ar^+^ ion beams) was used to compensate for the charging effect in the insulating samples. The most prominent transitions of the detected elements (O1s, Pt4f, Ce3d, Ti2p, and Si2p) on the outermost surface layer (<10 nm) were recorded in a high-resolution mode, and then they were deconvoluted.

The temperature-programmed reduction (H_2_-TPR) and H_2_ chemosorption experiments were carried out in a flow system with ChemBET TPR/TPD (Quantachrome, Boynton Beach, FL, USA), instrument apparatus equipped with thermal conductivity detectors (TCD). Some 50 mg of the powder catalyst was treated in a continuous flow of 5% volume H_2_ in Ar (70 mL min^−1^). The temperature was increased to 850 °C at a heating rate of 10 °C min^−1^.

XAS measurements were carried out at the 1W1B beamline at the Beijing (China) Synchrotron Radiation Facilities (BSRF), operating at 30–50 mA and 2.2 GeV, at room temperature. Due to the low mass concentration of Pt, XAS measurements were performed in fluorescence mode, using X-ray energy of incident flux between 11,370 and 12,430 eV.

### 2.4. Catalytic Activity Measurements

Catalytic oxidation of methane was performed using the flow set-up equipment, with a gas chromatograph with a thermal conductivity detector and a flow fixed-bed reactor. The chromatographic system had two analysis channels: Porapaq QS 80/100, and carbon molecular sieve columns. About 20 mg of catalyst was loaded into the quartz reactor tube (i.d. = 6 mm), which was placed in a tubular oven equipped with a temperature controller. Measurements were carried out within a 200–500 °C temperature range, with a volume ratio air/CH_4_ gas mixture of 2/1. The composition of the gas mixture was 10% CH_4_, 5% N_2_, and 85% Ar. The samples were stepwise heated with steps of 50 °C and maintained for 30 min at each level temperature, as described elsewhere [25].

## 3. Results

### 3.1. Properties of Titania Modified SBA-15 Supports

**Figure 1:**
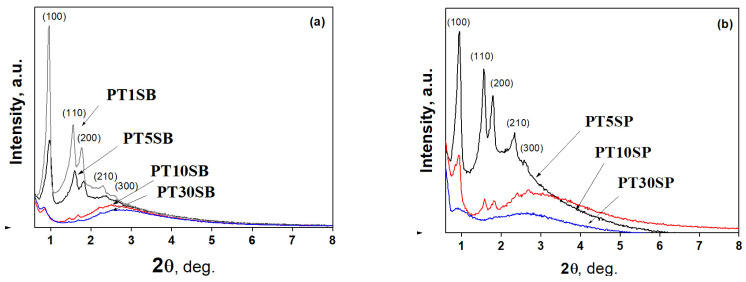
The low-angle XRD patterns of PtTi-SBA-15 samples with 1%, 5%, 10% and 30% TiO_2_ content obtained from TBOT (**a**) and PT (**b**).

**Figure 2:**
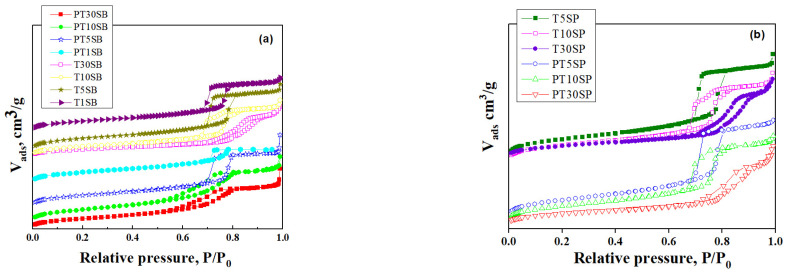
N_2_ adsorption–desorption isotherms of Ti-SBA-15 and PtTi-SBA-15 samples with varied TiO_2_ content obtained from TBOT (**a**) and PT (**b**).

The effects of the titanium precursor and its concentration on the ordered porous structure of the obtained PtTi-SBA-15 samples were investigated by small-angle X-ray diffraction and nitrogen adsorption–desorption (Figure 1 and Figure 2). XRD patterns of the PtTi-SBA-15 mesoporous silica supports exhibit five well-defined peaks in the range of 0.8 < 2θ < 3, due to (100), (110), (200), (210), and (300) reflections associated with perfect hexagonal (p6 mm) symmetry (Figure 1). The intensities of these peaks decrease with titania content and are very low for samples with 10 and 30% TiO_2_ content. All the samples preserved the ordered mesoporous structure of SBA-15, except for the PT30SP sample.

Nitrogen adsorption and desorption isotherms (Figure 2) can be classified as type IV, which are associated with mesoporous structures. The hysteresis loop corresponds to the H-1 type due to mesoporous SBA-15 silica. The Ti-SBA-15 supports with lower titania loading exhibit a high specific surface area (700–800 m^2^/g) and a large pore volume (1.1–1.4 cm^3^/g), with an average pore diameter between 6.4 and 7.2 nm. Conversely, richer titania content caused the specific surface area to lower to around 500 m^2^/g, with a pore volume of 1 cm^3^/g. Increasing the titania content to 10% determined a decrease in pore diameter, and for samples with 30% TiO_2_, a significant increase in the size of the pores was evidenced (8–9 nm). The increasing pore diameter for supports with 30% TiO_2_ is a result of porous structure degradation (Figure 1), which is more obvious for the sample obtained with peroxotitanate.

No significant variation in these parameters was observed after Pt immobilization. A narrow pore size distribution was obtained for the samples with lower titania content (Figure S1).

**Figure 3:**
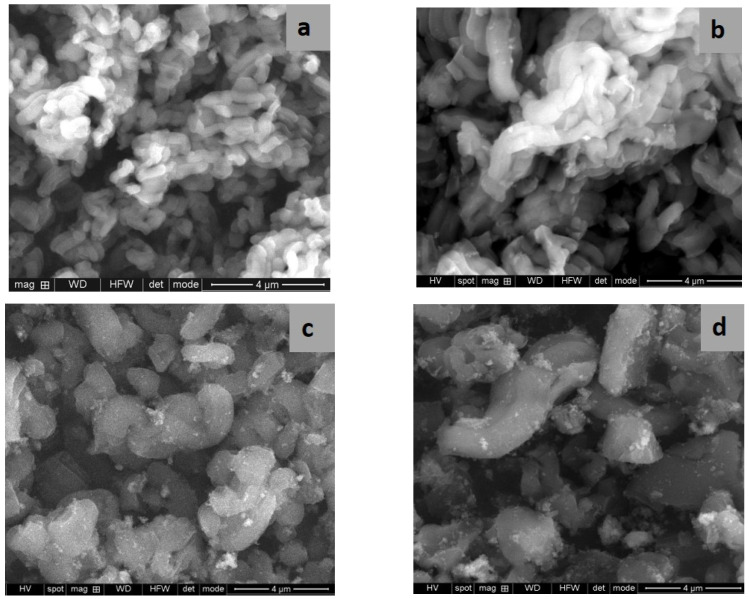
SEM images of PT1SB (**a**), PT5SB (**b**), PT10SB (**c**) and PT30SB (**d**) samples.

**Figure 4:**
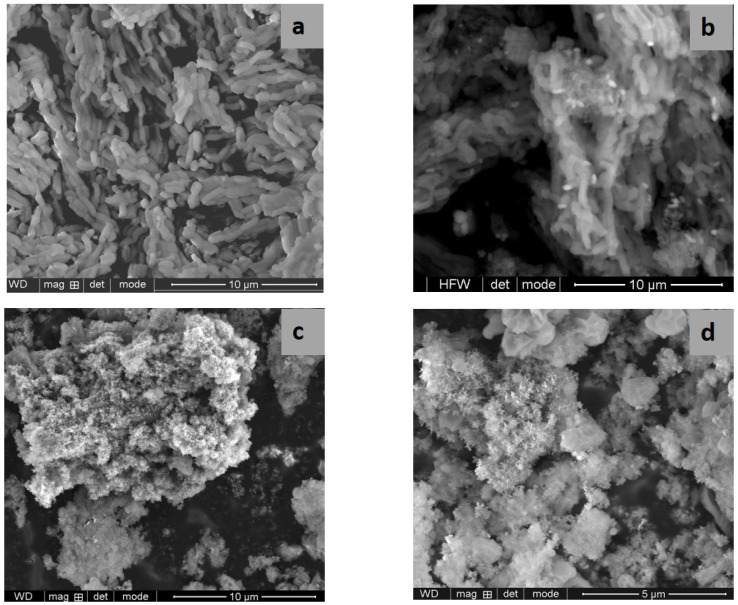
SEM images of PT5SP (**a**), PT10SP (**b**), and P30SP (**c**,**d**) samples.

**Figure 5:**
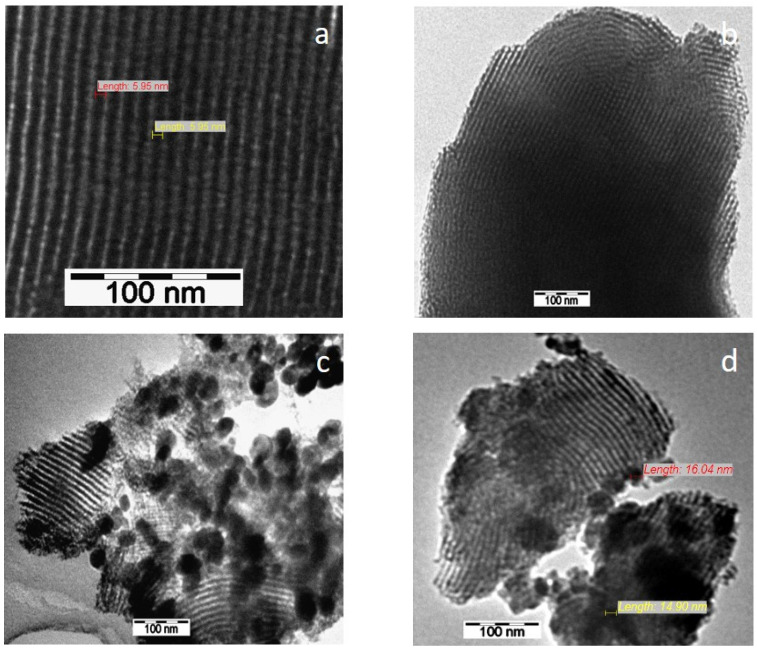
TEM images of PT1SB (**a**), PT5SB (**b**), PT30SB (**c**) and PT10SB (**d**) samples.

SEM and TEM images evidenced the effects of titania content and precursors on the characteristic ordering of PtTi-SBA-15 samples. A rod-like morphology is noticeable in Figure 3 for all the samples. At higher concentrations of TiO_2_, another phase deposited on the surface of the silica particles can be observed. At low concentrations of TiO_2_, the species of Ti are in the SBA-15 network or on the surface, without being able to be highlighted as a distinct phase, which is observed in the case of higher titania loading (Figure 4c,d). For the peroxotitanate-derived samples, the SEM images (Figure 4a,b) also indicate a rod-like morphology for low concentrations of TiO_2_. A flower-like morphology became dominant when the titania concentration increased to 30% (Figure 4c,d). SEM images recorded using a back-scattered electron (BSE) detector were presented in order to evidence Pt dispersion on the surface, using the advantage of the compositional contrast (Figure S2). It is obvious that the concentration of Pt increases with the increase in titania content. The homogeneous dispersion of Pt and Ti components in the silica network for samples with lower titania content is noticeable in the SEM elemental mapping of the microstructure (Figure S3). These images confirm the existence of Ti species and the possibility of their agglomeration on the silica surface at higher titania contents. An ordered hexagonal porous structure was highlighted in TEM images of samples obtained from TBOT (Figure 5).

### 3.2. Physicochemical Properties of the Supported Metals Species

**Figure 6:**
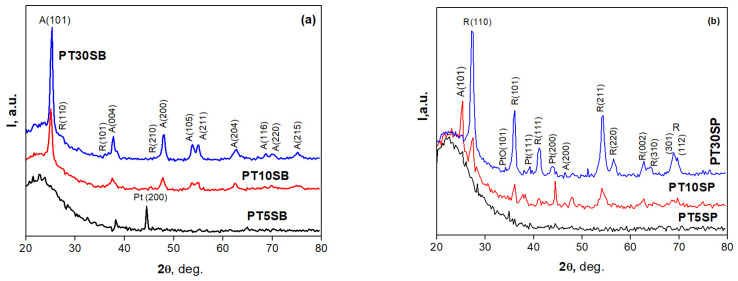
The wide-angle XRD patterns of PT1nSB (**a**) and PTnSP (**b**) samples.

Wide-angle XRD patterns (Figure 6) indicate distinct TiO_2_ crystalline peaks attributed to anatase and rutile. Unfortunately, the very low concentration of Pt (0.25% wt.) and the possibility of the Pt and TiO_2_ species peaks overlapping do not allow a very accurate interpretation of the results. Taking into account the concentration of TiO_2_ in the samples, it can be considered that most of the peaks correspond to titanium species. The intensity of the TiO_2_ crystalline peaks increases upon increasing its loading. This increase is more evident for the peroxotitanate-obtained samples with a richer rutile phase.

UV-Raman spectroscopy is a powerful technique for the surface characterization of mesoporous catalysts, while VIS-Raman spectroscopy mainly addresses the structural investigation of non-framework titania, and less so mesoporous catalyst support [41,42].

**Figure 7:**
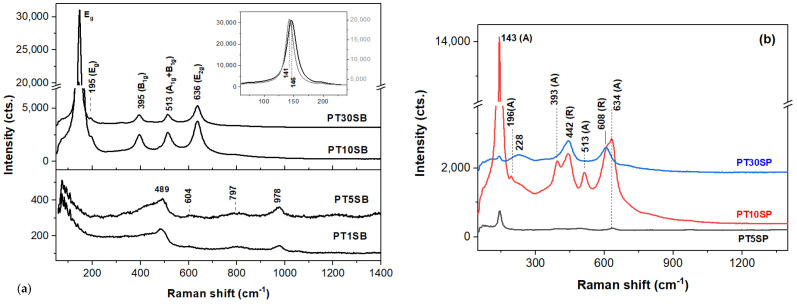
VIS-Raman spectra of PTnSB (**a**) and PTnSP (**b**) samples (A and R stand for anatase and rutile).

Since platinum is not Raman-active, VIS-Raman spectra of the Pt-containing materials in Figure 7 are due to TnS(B/P) materials and platinum oxides. Bands located at about 489, 604, 797, and 978 cm^−1^ originate from four- and three-membered SiO_4_ rings, and the Si-O-Si bending and Si-O stretching vibrations [43] of the SBA-15 (Figure 7a) point out that the framework titanium species (TiO_4_ units) [42,44] are present in the PT1SB and PT5SB samples with low TiO_2_ content. This finding is supported by the UV-Raman band at 1089 cm^−1^ (stretching vibrations of the Si-O-Ti bonds [44]) for the PT5SB sample, while the 1057 cm^−1^ band of the PT1SB spectrum in Figure S4 a is attributable to an asymmetric stretch of Si-O linkages [22]. Li et al. assigned the 490, 530, 960, and 1125 cm^−1^ bands collected with a deep UV laser (244 nm) to Ti-O-Si linkages [44]. However, the latter band is almost undetectable for the 325 nm-excited spectra [44]. This is analogous to the UV-Raman spectrum of the PT5SP catalyst (Figure S4b), wherein the 490, 530, and 960 cm^−1^ bands are more obvious. Moreover, Pt-O stretching modes were reported to appear at about 550 cm^−1^ and 532 cm^−1^ in the UV-Raman spectra of the platinum-rich loadings (3%), SBA-15, and platinum powder, respectively [43].

The intensity of the two defect bands at 489 (D_1_) and 604 cm^−1^ (D_2_) is sensitive to the presence of HO^-^ and the porous silica network with low TiO_2_ loads. The relatively intense band at ~3740 cm^−1^ (isolated SiO-H, H-bonded) in the UV-Raman spectra in Figure S4 confirms that OH groups are linked to the SBA surface [45]. Thus, the intensity of the D_2_ band decreases and vanishes when the porous structure degrades and/or collapses. Among the low TiO_2_ content samples, the PT5SP spectrum in Figure S4b lacks the D_2_ band. Since no significant modification of the surface area for the two PT5S(B/P) is recorded, the missing D_2_ band is a consequence of the overwhelming 634 cm^−1^ band of anatase [46] present in the VIS- and UV spectra (Figure 7 and Figure S4b).

Anatase vibrational modes [46] at 146 cm^−1^ (E_g_), 195 cm^−1^ (E_g_), 395 cm^−1^ (B_1g_), 513 cm^−1^ (A_1g_ + B_1g_), and 639 cm^−1^ (E_g_) are identified for higher TiO_2_-loaded samples, i.e., PT10SB and PT30SB, respectively (Figure 7a). The shifting of the E_g_ mode (146 cm^−1^) towards lower wavenumbers (see inset of Figure 7a) when the TiO2 content increases to 30 wt. % (PT30SB), might signal defects and/or nonstoichiometry of the anatase phase [47]. In contrast to PT5SB, the VIS-Raman spectrum of the PT5SP indicates the presence of anatase through its main bands at 143 and 636 cm^−1^ (Figure 7b). The relatively reduced intensity of the E_g_ band at 143 cm^−1^ is a consequence of diminished anatase content. Although obscured by strong bands of anatase, weak bands of SBA-15 at 489, ~800, and 977 cm^−1^ are still present in the VIS-Raman spectrum of the PT5SP.

Increasing the TiO_2_ content triggers the formation of the rutile [46] (143, 447, 612, and 826 cm^−1^ of the B_1g_, E_g_, A_1g_, and B_2g_ modes, respectively) and anatase phase. Zhang et al. [47] reported an anatase–rutile transition at lower temperatures when the initial particle size is reduced. The wide band at 228 cm^−1^ is due to second-order and disorder effects [48]. When TiO_2_ increases to 30 wt.%, the TiO_2_ prevailing phase is rutile. It is very likely that the anatase and rutile phases are distributed in so-called core–shell structures [49,50,51]. Hence, the non-framework titania (TiO_6_ clusters), namely anatase and/or rutile, is formed in the titania-rich PTnS(B/P) catalysts.

**Figure 8:**
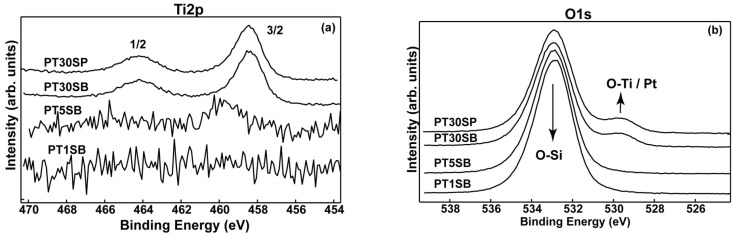
The XPS photoelectron spectra: Ti2p (**a**) and O1s (**b**) superimposed spectra for PT1SB, PT5SB, PT30SB and PT30SP.

Analysis of the catalyst surface composition was carried out using XPS spectroscopy. Figure 8 displays the high-resolution XPS spectra of Ti2p and O1s, and Figure S4 shows the Si2p species detected on the surface of the PtTi-SBA-15 samples. The binding energies (Bes) of the Ti 2p doublet (2p3/2 = ~458.4 eV and 2p1/2 = 464.6 eV) reveal the presence of the Ti^4+^ cations in the TiO_2_ lattice (see Figure 8a) for the PT30SB and PT30SP samples. We assigned the higher BE (459.8 eV) in the sample PT1SB, with a small amount of Ti, to the stronger interaction of Ti with SiO_2_ support, as found by Lassaletta et al. [52]. Thus, the Ti oxidation state is 4+, as usual, but is shifted toward a higher BE due to the aforementioned interaction. Table 1 shows a low concentration of titanium atoms on the surface. One can see that in the sample PT1SB, Ti was not detected on the top of the surface (the first ~10 nm). In the case of titanium immobilization using direct synthesis on a mesoporous silica support, such as SBA15, the substitution of silica in the network takes place. For very low concentrations, Ti can be totally incorporated into the network [53]. This is evidenced by the high dispersion of titanium in the silica network for the samples with lower TiO_2_ percent, and the agglomeration of crystalline oxide on silica particles for samples with higher titania loading.

The effect of the support and synthesis method on extra-framework titanium species has been highlighted by previous studies [24,25]. It was thus observed that the XRD results either indicated or not, for the same concentration of TiO_2_ (5%), the presence of its crystalline species. At the same time, the XPS spectra showed different concentrations of Ti on the surface under the conditions of the same TiO_2_ concentration in the sample. Thus, the different distribution of titanium in the volume compared to the surface was highlighted. Additionally, the concentration of titanium oxide and its association with other oxide species, such as those of cerium, influenced the concentration and nature of the Pt species on the surface.

**Figure 9:**
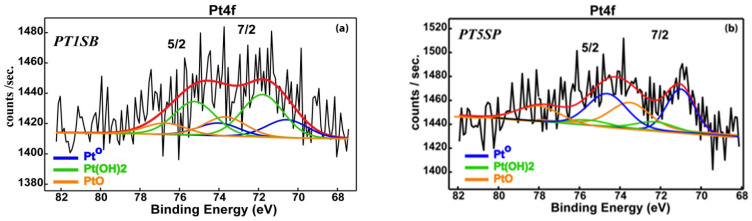

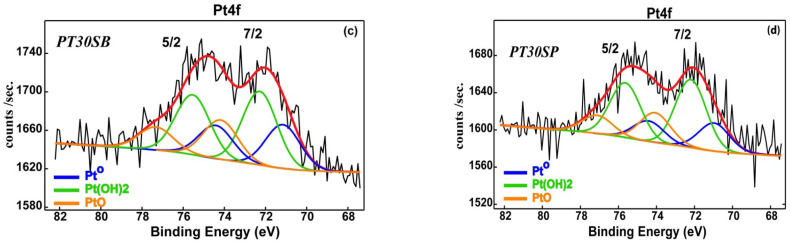
XPS spectra of PtTi-SBA-15 samples with (**a**) 1, (**b**) 5, and (**c**,**d**) 30% TiO_2_ loading. (Red line stands for global fit).

The O1s spectra (Figure 8b) show a similar profile for the samples PT30SB and PT30SP with oxygen mainly bonded in SiO_2_ matrix (532.9 eV), as well as a small contribution assigned to oxygen bonding with titanium (530.4 eV). The Si^4+^ oxidation state was found in all samples and confirmed by the Si2p transition at 103.4 eV (Figure S5). Pt4f spectra exhibit a complex band-like shape, accommodating elemental Pt and Pt oxidized as Pt (OH)_2_ and PtO (Figure 9). Despite very low Pt concentrations, and consequently, associated noisy spectra, it is still possible to deconvolute Pt4f spectra in an attempt to determine the Pt chemical species. The deconvoluted procedure for current Pt 4f noisy spectra followed ISO-TC201 (“surface chemical analysis”) recommendations, using the constraints on the binding energies (Bes) associated with Pt^0^ (4f7/2 = 71.1 eV), Pt(OH)_2_ (4f7/2 = 72.2 eV)) and PtO (4f7/2 = 74.4 eV), the intensity ratio in the 4f7/2, 5/2 doublet, and the spin–orbit parameter. It is worth mentioning that the errors in the binding energies (Bes)’ assignments were within ±0.2 eV limits. These led to a reliable quantitative assessment, even for the spectrum with very low count rates, as a result of tiny relative concentrations (Table 1). Thus, one can see that samples PT30SB and PT30SP display about the same Pt oxide and metal concentration.

A close examination of the spectra suggests a larger contribution of the elemental Pt to the PT5SB sample (57.4% Pt^0^) as compared to the PT1SB sample (22.6% Pt^0^).

**Figure 10:**
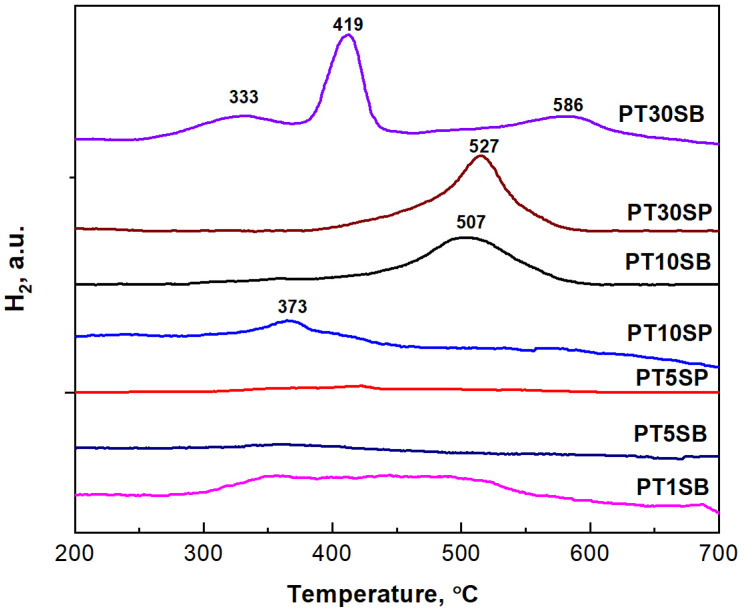
H_2_-TPR profiles of PTnSB and PTnSP catalysts with different TiO_2_ loading.

The redox properties of the PtTi-SBA-15 catalysts, after their thermal treatment in air at 550 °C, are shown in Figure 10. High-temperature reduction peaks in the range of 300–600 °C can be observed. This is in agreement with the XPS spectra of the other Pt/TiO_2_ catalysts [54,55]. The low-temperature peaks were attributed to the reduction of PtOx species, whereas those appearing at high temperatures were associated with the reduction of TiO_2_, catalyzed by Pt through the strong metal–support interaction (SMSI). Three reduction peaks were observed for sample PT30SB, and one broad peak for all other samples. The first was attributed to the reduction of the PtO_x_ species. The next two may be the result of two different Ti oxide species.

The reduction of TiO_2_ species occurs at 419 °C, with a high interaction with Pt, and at 586 °C, titania is reduced, with a higher interaction with the silica support. The interaction of Pt with the supported TiO_2_ explains the hydrogen absorption at a higher temperature, e.g., 507 °C (PT10SB) and 527 °C (PT30SP). Previous studies [56,57] have also indicated that between 300 °C and 400 °C, Ti^4+^ to Ti^3+^ can be partially reduced by hydrogen spillover in the presence of Pt nanoparticles, and after 500 °C, the final reduction of the titanium species takes place.

In both cases, there is a significant effect caused by SMSI, and a spillover of dissociative hydrogen is chemisorbed [58]. The more evident spillover effect was observed for the PT1SB sample, with a large peak between 350 and 550 °C. At a low concentration of TiO_2_ (1%), it was proven that in the case of direct synthesis, Si-O-T-O-Si species are formed in the silica network. In this case, the probability of Pt-TiO_2_ interaction is negligible. In this case, the platinum is deposited on the silica. Therefore, platinum interacts with silica in the PT1SB. The broad peaks at around 350 °C were assigned to the reduction of PtO species and to the H_2_ spillover effect. This peak was very small for some samples, namely PT5S(B/P), with a higher Pt metal content and lower consumption of H_2_ (Table 2).

The Pt particles’ nature, especially their size, and dispersion, was analyzed using the H_2_ chemisorption method. High dispersion of Pt, with an average size of around 1 nm, was obtained for all the samples (Table 2). The amount of hydrogen consumption needed for reduction was higher for samples with larger peaks, such as PT1SB, PT10SB, or PT10SP. All these samples have one peak of reduction as a result of both the SMSI effect and H_2_ spillover.

**Figure 11:**
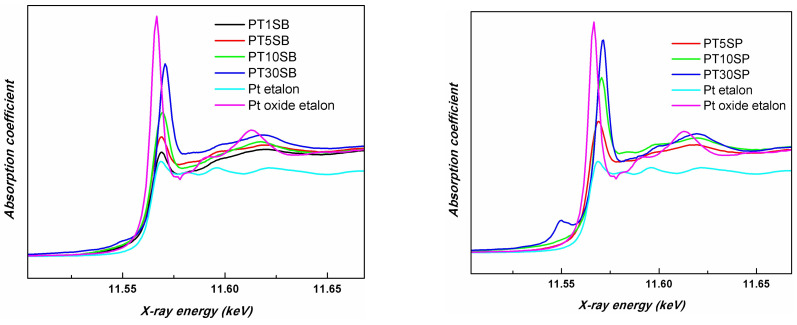
Pt LIII-edge XANES spectra of investigated samples together with the etalon samples.

The X-ray absorption near edge spectroscopy (XANES) measurement results confirm the presence of Pt in both metallic and oxidation states. The Pt LIII-edge XANES spectra of the investigated samples together with the etalon samples are presented in Figure 11. The absorption occurring at the Pt LIII-edge corresponds to the 2p3/2→5d electronic transitions [59,60]. The energy shift observed in the Pt LIII-edge XANES spectra is not sufficiently large to distinguish between the Pt oxidation states. This is the reason that the intensity of the white line peaks was also considered. Thus, the white line intensity reflects the unoccupied Pt(5d) orbital because the absorption edge corresponds to an electron transition from the 2p3/2 to 5d orbitals in the Pt atom [61].

**Figure 12:**
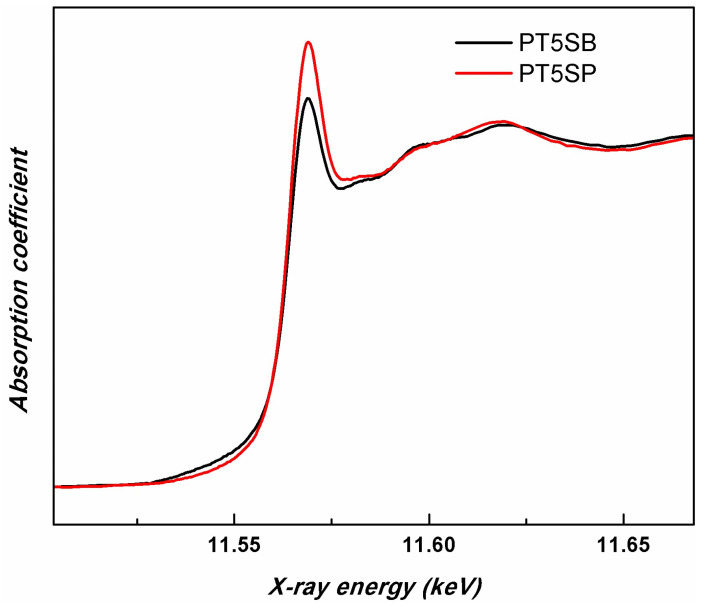
Pt LIII-edge XANES spectra of PT5SB and PT5SP samples.

In order to compare the different support influences (TnSB and TnSP), the Pt LIII-edges of the samples with the same concentration of TiO_2_, namely PT5SB, and PT5SP, were plotted in Figure 12. The intensity of the white line is higher for the PT5SP sample. Moreover, the formation of the non-framework titania threshold is lowered to 5% in the case of the SP support, as supported by the XRD and Raman findings (Figure 6b and Figure 7b), wherein anatase was depicted.

### 3.3. Catalytic Properties of PtTi-SBA-15 Materials in CH_4_ Oxidation

**Figure 13:**
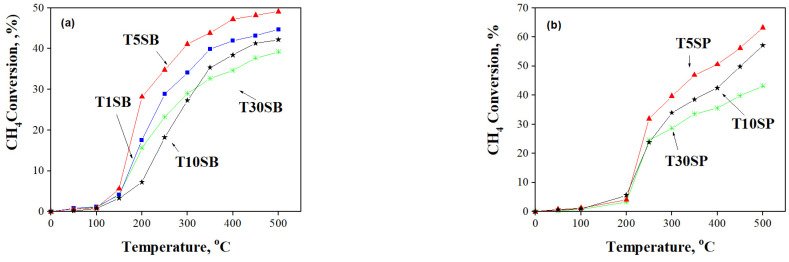
CH_4_ conversion as a function of temperature for (**a**) TnSB and (**b**) TnSP supports with different titania loadings.

**Figure 14:**
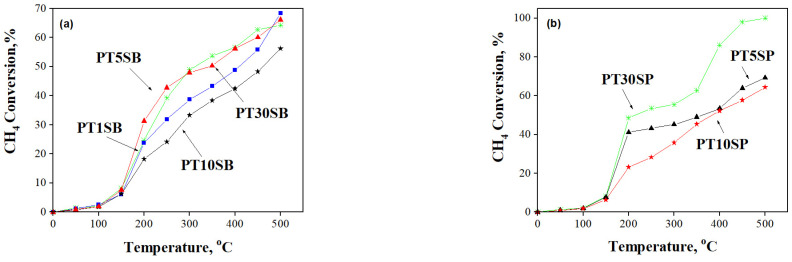
CH_4_ conversion and its variation with temperature for (**a**) PTnSB and (**b**) PTnSP samples after Pt immobilization.

The methane oxidation occurring in Ti-SBA-15 supports and Pt-based catalysts was monitored as a function of temperature (Figure 13 and Figure 14). All the samples are active in CH_4_ oxidation reactions, and a higher selectivity to CO_2_ was obtained for samples immobilized with Pt (>90%).

All TnSB and TnSP supports with 1, 5, 10, and 30% TiO_2_ content are active in methane oxidation (Figure 13). High activity was obtained for TnSB samples after Pt immobilization (Figure 14). The highest conversion of methane was obtained above 400 °C for the sample prepared from peroxotitanate with 30% TiO_2_.

## 4. Discussion

Two different Ti-SBA-15 supports were prepared using the direct synthesis method. One was obtained from tetra butyl ortho-titanate (TBOT) as a titanium dioxide precursor [22,24], and the other from peroxotitanate synthesized from TBOT and H_2_O_2_. The nature and concentration of the titanium precursors influenced the sol–gel process in the presence of surfactant, and thus the formation of a silica network and titania crystalline species. The addition of TBOT in combination with TEOS to the aqueous solution of surfactant reduced the rate of titanium alkoxide hydrolysis in order to obtain its advanced dispersion within the silica species. The increasing TBOT concentration led to the growth of the hydrolyzed alkoxide species, and their agglomeration thus blocked the polycondensation of the silica. Thus, the TBOT precursor influenced the morphology and porous structure of the SBA-15 silica. After hydrothermal treatment and calcination, the presence of non-framework TiO_2_ crystals on the silica surface was detected for the titania-rich samples (Figure 6).

In the second synthesis method, the precursor of titania was peroxotitanate. The peroxo groups (O-O) were found either to accelerate photocatalytic activity through superoxide generation or to shift the catalytic activity to visible light due to oxygen vacancies [62]. The peroxotitanate route used in the synthesis of the silica–titania composite, using the sol–gel method with small TiO_2_ loadings, led to interesting properties; this is due to the formation of Si-O-Ti linkages and small crystalline anatase particles because silica suppresses the growth and aggregation of TiO_2_ crystals. A well-crystallized rutile phase was obtained using the sol–gel method and hydrothermal peroxo route at high TiO_2_ loadings. Thus, the Ti-hydroperoxy (OH)_4_-n-Ti(OOH)n species formed during preparation from TBOT and H_2_O_2_ in an aqueous solution further dissociated into protonated Ti-peroxo species (OH)_4_-n-Ti(OO-)n. Chung et al. [63] reported lower free energies for the anatase overall reaction than in the rutile reaction in TiO_2_ catalysts. The influence of these anions on the formation of the silica network is smaller in an acidic solution of surfactant and TEOS silica precursor. However, they can be adsorbed on the surface of the silica at small concentrations and may form aggregates of rutile when the concentration of titania is higher. The formation of rutile nanocrystals was considered the result of hydrothermal treatment and of the TBOT/H_2_O_2_ molar ratio.

The presence of different titania polymorphs and Pt species, the change in the morphology (Figure 4c,d and Figure 5c,d), and the diminishing of the silica-ordered structure was evidenced for the samples with 10 and 30% TiO_2_ (Figure 6c,d). XRD patterns (Figure 1), SEM (Figure 3 and Figure 5), and TEM (Figure 5) images show the significant effect of the titanium precursor and its concentration on the morphology and porous structure of PtTi-SBA-15 samples, which is more significant in the case of SBA-15 compared to KIT-6 [25]. Although TBOT was the titanium precursor for both synthesis methods, the type of the non-framework titanium dioxide polymorph was determined by the SBA-15 and KIT-6 supports as well as the TBOT and PT precursors. Thus, direct synthesis of Ti-SBA-15 favored the attainment of anatase and rutile phases. The concentration of these species increased significantly in the case of peroxo-synthesized catalysts (PTnSP materials). According to Raman data in Figure 7, the titania-rich sample PT30SP contains mostly rutile phase in comparison with the PT30SB counterpart in which the anatase phase prevails. Hence, when using the peroxo route, the non-framework rutile phase of titania formed at high titania loadings. Moreover, the TiO_2_ content threshold is lowered to 5% in the case of the PTnSP, in which the non-framework anatase prevailed.

XPS results showed the presence of Pt^0^ species on the surface. Their content decreased for the samples with higher titania loadings. A very low effect of the titania precursor was evidenced for the superficial Pt species. Comparatively, with PtTi-KIT-6 samples, XPS of PtTi-SBA-15 samples evidenced a lower percentage of Pt on the surface under conditions of samples with the same composition [25]. These differences are caused by changes in morphologies, porous structures, the dispersion of titanium species, and their interaction with the supports.

H_2_-TPR results evidenced the interaction of Pt species with supports of different compositions that modified PtO_x_’s reducibility to Pt^0^ [64,65]. Different degrees of the metal–support interaction was also observable in the TiO_2_ reduction pattern above 400 °C [66]. The strong interaction of Pt with the TiO_2_ substrate is attributable to an electron transfer from the substrate to Pt atoms. However, with the increase in TiO_2_ concentration, this transference is reduced, and for the sample with higher TiO_2_ content, the Pt oxide concentration increases. This phenomenon is a result of TiO_2_ crystalline phase agglomeration with the increase in its concentration, which causes a decrease in Pt-TiO_2_ surface interaction (Figure 4 and Figure S2). The small differences between XPS and XAS results concerning the relative concentration of Pt species can be explained by considering that XPS provides surface information, while XAS is a volume technique. Therefore, the presence of Pt^0^ in all the PTnS samples sustains the metal–support interaction. However, the interaction between Pt and titania depends on titania’s dispersion on a mesoporous silica support, its particle size, and its crystalline structure.

The best conversion was obtained for samples with the highest dispersion and an insignificant crystalline phase of titania (see Figure 2 and Figure S2). A higher CH_4_ conversion was obtained for PTnSB samples with 5% and 30% TiO_2_ concentrations, respectively. This result was explained by the SMSI effect evidenced both in the reduction of PtO to Pt^0^, at a higher percentage for sample PT5SB and at a higher Pt percentage on the surface of the PT30SB sample, as determined by XPS (Table 1). The Pt/PtO ratio of the atomic species (Table 1) is 1.35 for the PT5SB sample and 0.43 for the PT30SP sample. Parmon et al. [67] showed using XPS measurements that the catalyst activation in methane oxidation may be associated with the predominant formation of the metallic platinum, whereas the oxidation of the platinum surface is responsible for the less active state. This effect of the metal oxidation state (Pd^2+^/Pd^0^ ratio) on activity in the total oxidation reaction of methane was evidenced for Pd-Pt/Al_2_O_3_ catalysts in situ XPS experiments [68]. The presence of Pt on the surface influenced the Pd^2+^/Pd^0^ ratio and hence the activity. The literature data indicate that only the coexistence of Pd^0^ and Pd^2^+ determines the high values of the activity in the total oxidation of methane or propane [69]. In this study, the conversion obtained for the PT30SB sample was considered the result of SMSI, in which the effect of the Pt/PtOx value is offset by increasing the Ti and Pt species on the surface. Thus, catalysts with 5% TiO_2_ may be considered the best catalysts. The catalyst PT30SP exhibited more effective conversion, reaching the maximum value (100%) at 500 °C, while SMSI had a lesser effect on the samples, with a maximum percentage of rutile on a silica support. The published studies [66] have shown that the crystalline composition of anatase and rutile TiO_2_ can affect the interaction with the support, the dispersion, and the oxidation state of the metal oxide support, i.e., PtO. For the PT30SP sample, rutile provides large amounts of oxygen atoms for reaction with methane. In the presence of higher Pt atoms on the surface, a great conversion of methane was achieved. Increased conversion at higher temperatures was supported by the catalytic test results and TPR data. Thus, the H2-TPR results showed the possibility of reducing oxide metal species at higher temperatures. In this way, taking methane as a reducing agent, the increase in conversion with temperature can be explained.

## 5. Conclusions

Two series of catalysts with highly dispersed Pt nanoparticles were prepared on Ti-modified SBA-15 mesoporous silica. SEM, XPS, XAS, TPR, and H_2_ chemisorption measurements revealed changes in Pt-TiO_2_ interaction with titanium species’ dispersion on the silica support and its crystalline structure. The properties of the PTnS(B/P) are a consequence of the synthesis conditions and titania loading. The catalysts exhibited a different concentration of Pt^0^ nanoparticles resulting from the stronger interaction of PtO with anatase and the weak interaction of PtO with rutile TiO_2_. Titania loading and precursors influenced the anatase and rutile concentration, the dispersion of titania species, and the Pt-TiO_2_-supported interaction. The results of the catalytic tests highlighted the synergistic effect of Pt and TiO_2_ species. The Pt/PtO ratio and their concentration on the catalyst surface, trigged by the SMSI effect, also influenced methane conversion. The increase in TiO_2_ concentration led to an increase in Pt species on the surface, which may explain the high conversion value of the PT30SB and PT30SP samples. In the case of the PT5SB sample, the high conversion value may be due to the higher percentage of metallic Pt dispersed on the surface. The best conversion (100%) was obtained for the PT30SP sample at 500 °C, and this may be the result of the Pt’s interaction with more crystalline TiO_2_ and a richer rutile phase. Although the impact of the TiO_2_ crystalline phase on catalytic performance was less known, here, its beneficial effect on methane oxidation has been evidenced.

## Figures and Tables

**Table 1:** XPS data for the catalysts (atomic relative concentrations, and percentages of Pt chemical species).

	Pt chemical Species, % (Pt4f7/2 Bes)			Atomic Relative Concentration, at%			
	Pt^0^ (71.1 ± 0.2 eV)	PtO (72.2 ± 0.2 eV)	Pt(OH)_2_ (74.4 ± 0.2 eV)	O	Si	Ti	Pt
PT1SB	22.6	22.2	55.2	71.40	28.45	0.13	0.02
PT5SB	57.4	8.4	34.2	71.58	28.22	0.17	0.03
PT30SB	30.2	21.2	48.6	71.69	25.42	2.84	0.06
PT30P	27.2	17.7	55.1	71.47	26.11	2.38	0.04

**Table 2:** H_2_ chemosorption results.

Catalysts	Dispersion, %	Average Crystallite Size, nm	Metal Surface Area, m^2^/g	H_2_ Consumption, µmol/g
PT1SB	26.7	1.2	69.6	169
PT5SB	45.8	0.84	112.7	48
PT10SB	41.3	0.95	102.5	124
PT30SB	38.1	0.98	94.06	86
PT5SP	48.1	0.78	118.5	50
PT10SP	43.5	0.99	98.6	145
PT30SP	32.5	1.16	79.95	88

## Data Availability

The data presented in this study are available on request from the corresponding author.

## Supplementary Materials

The following supporting information can be downloaded at: https://www.mdpi.com/article/10.3390/nano13101647/s1. Figure S1: Pore size distribution of PtTi-SBA-15 samples, before and after Pt immobilization on samples obtained with tetrabutylorthotitanate; Figure S2: Pore size distribution of PtTi-SBA-15 samples, before and after Pt immobilization on samples obtained with peroxotitanate; Figure S3: Elemental mapping images obtained by SEM; Figure S4: UV-Raman spectra of the PT(1/5)S(B/P) catalysts. Figure S5: The XPS photoelectron spectra of Si2p, superimposed spectra for PT1SB, PT5SB, PT30SB and PT30SP.
