# Supplementary material for: Variation in Metal–Support Interaction with TiO2 Loading and Synthesis Conditions for Pt-Ti/SBA-15 Active Catalysts in Methane Combustion
Source: Nanomaterials (Basel). 2023 May 15;13(10):1647. doi: 10.3390/nano13101647 (PMC10223147; doi:10.3390/nano13101647)
Supplement: Supplementary file 1 [file nanomaterials-13-01647-s001.zip › nanomaterials-2349241-supplementary.pdf]

Variation of Metal-Support Interaction with TiO_2 loading and synthesis conditions for Pt-Ti/SBA-15 active catalysts in methane combustion

Mihaela Filip[1], Elena Maria Anghel[1,*], Vasile Rednic[2], Florica Papa[1], Simona Somacescu[1], Cornel Munteanu[1], Nicolae Aldea[2], J. Zhang [3], and Viorica Parvulescu[1,*]

[1] Ilie Murgulescu Institute of Physical Chemistry, Romanian Academy, Spl. Independentei 202, 060021 Bucharest, Romania
[2] National Institute for R&D of Isotopic and Molecular Technologies, Donat St. 67-103, 400293, Cluj-Napoca, Romania
[3] Beijing Synchrotron Radiation Facilities of Beijing Electron Positron Collider National Laboratory,19B Yuquan Road, 100049, Beijing, China
* Correspondence: vpirvulescu@icf.ro, manghel@icf.ro

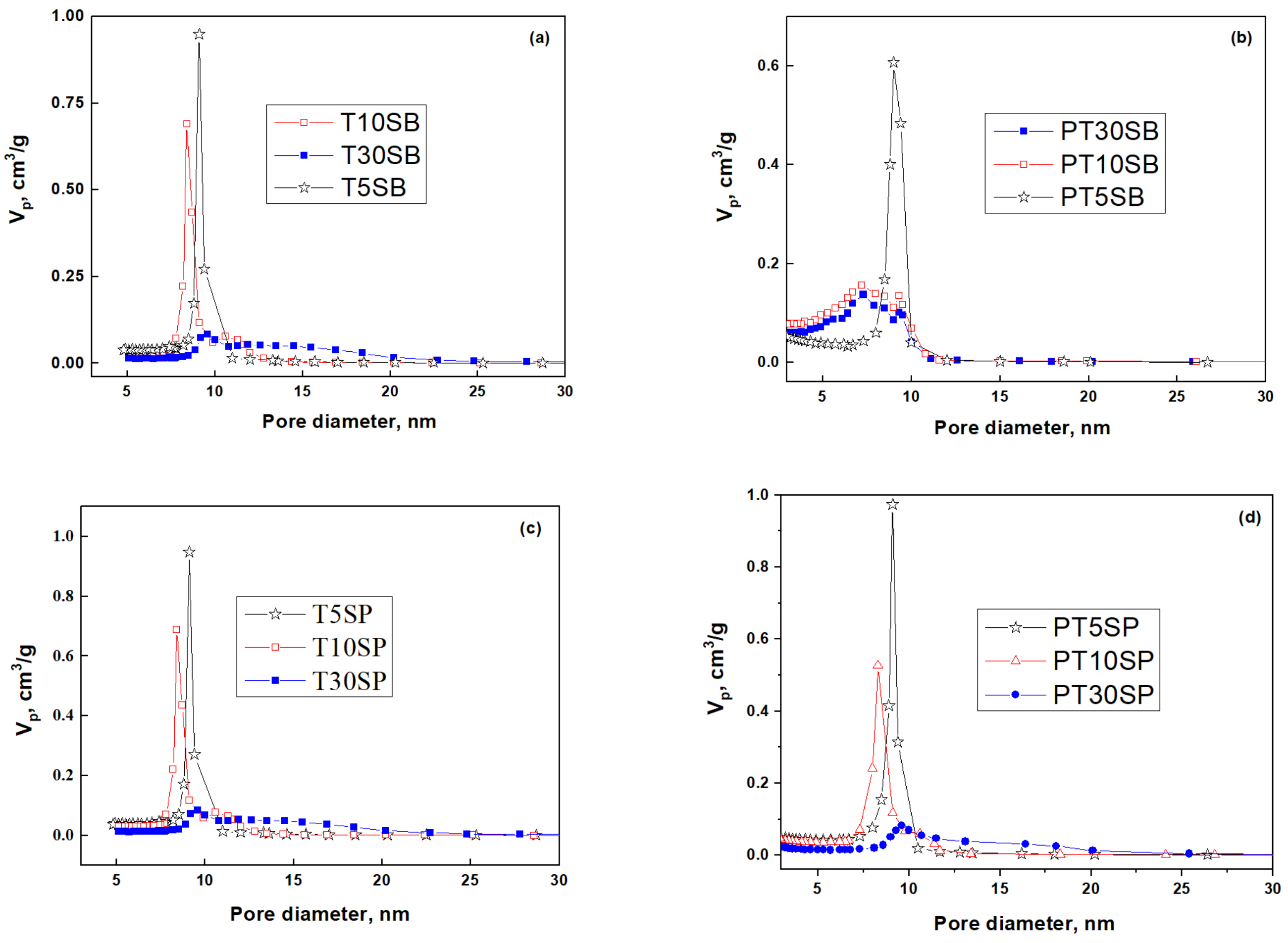

Figure S1. Pore size distribution of the (-/Pt)Ti(5/10/30)SB samples, before (a) and after Pt (b) immobilization on samples obtained with tetrabutylorthotitanate, and the (-/Pt)Ti(5/10/30)SP samples, before (c) and after Pt (d) immobilization on samples obtained with peroxotitanate.

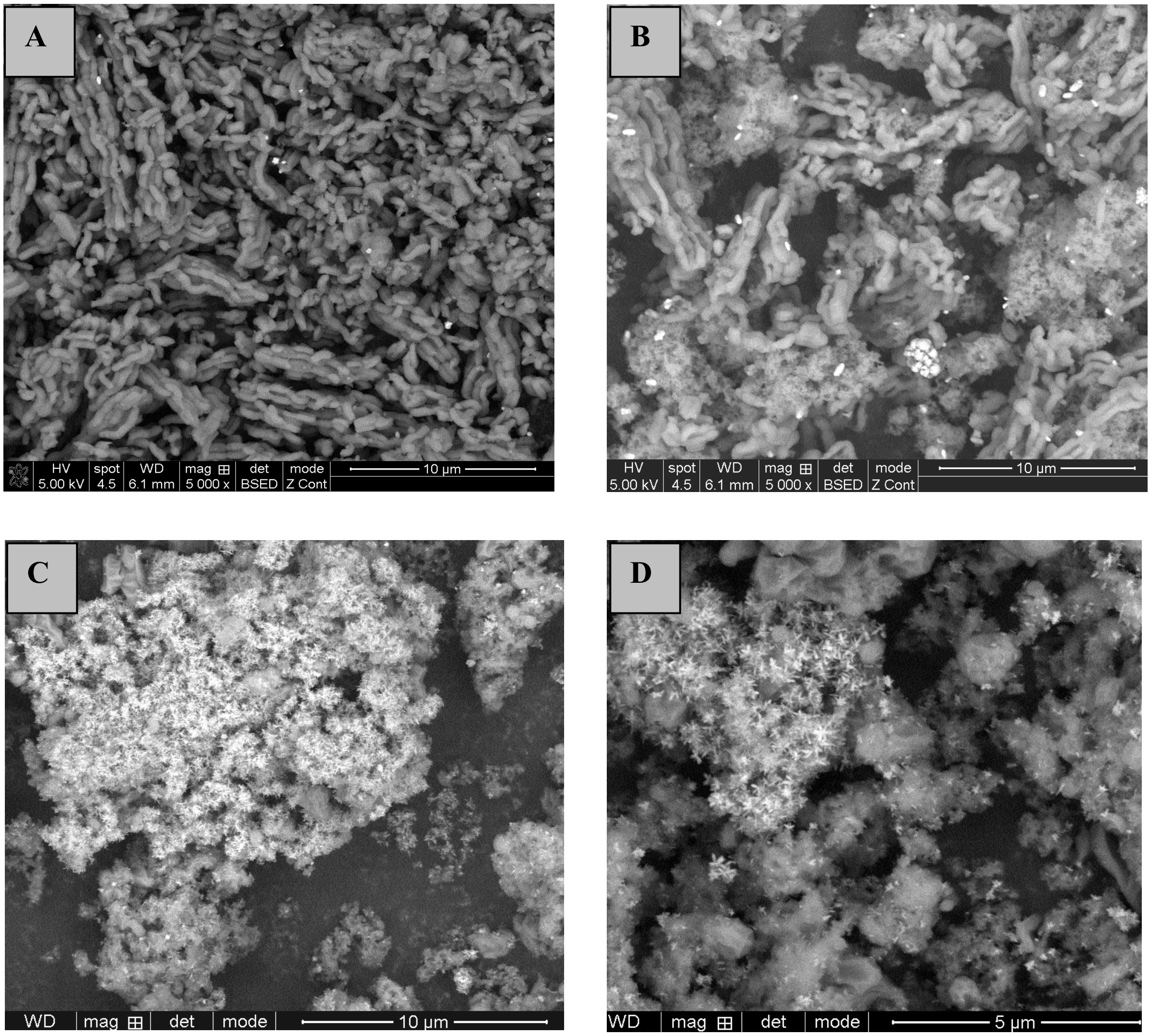

Figure S2. SEM images, recorded using back-scattered electrons (BSE), of PT5SP (A), PT10SP (B) and P30SP (C, D) samples.

PT1SP

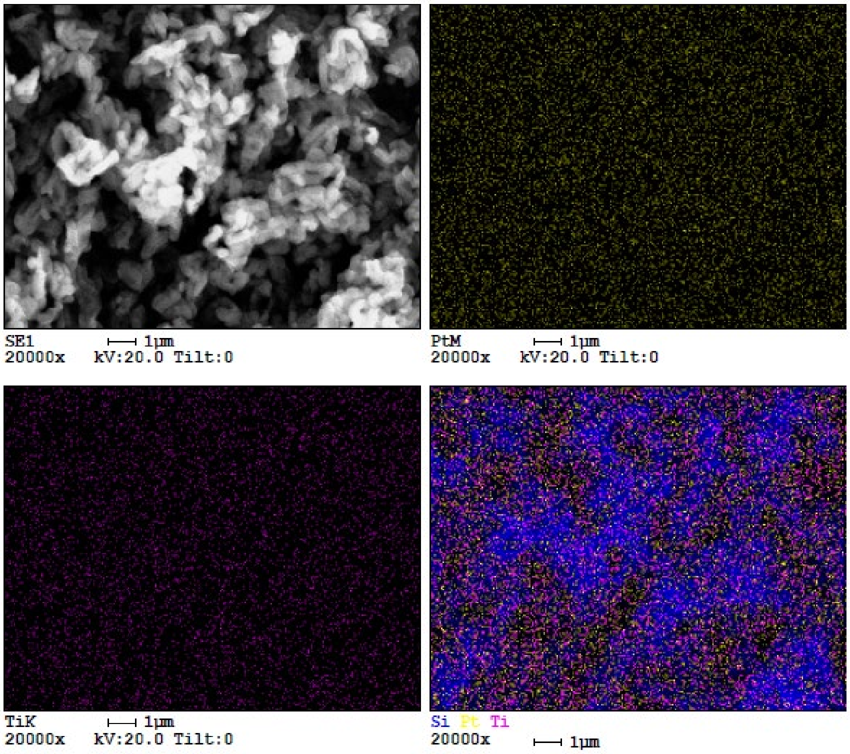

SE1 1µm
20000x kV:20.0 Tilt:0
PtM 1µm
20000x kV:20.0 Tilt:0
TiK 1µm
20000x kV:20.0 Tilt:0
Si Pt Ti
20000x 1µm

PT5SP

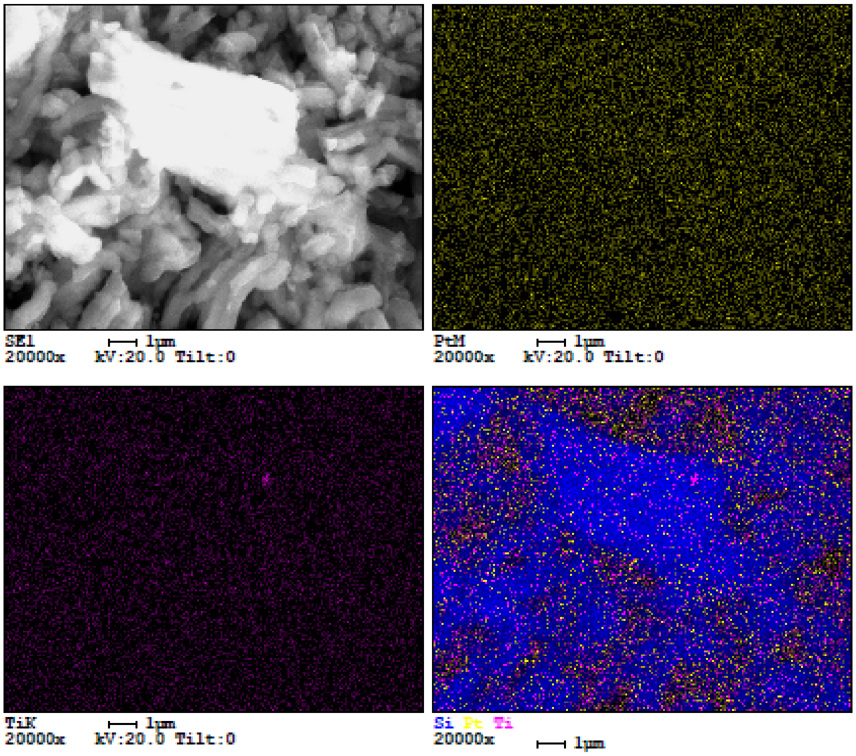

SE1 1µm
20000x kV:20.0 Tilt:0
PtM 1µm
20000x kV:20.0 Tilt:0
TiK 1µm
20000x kV:20.0 Tilt:0
Si Pt Ti
20000x 1µm

PT10SB

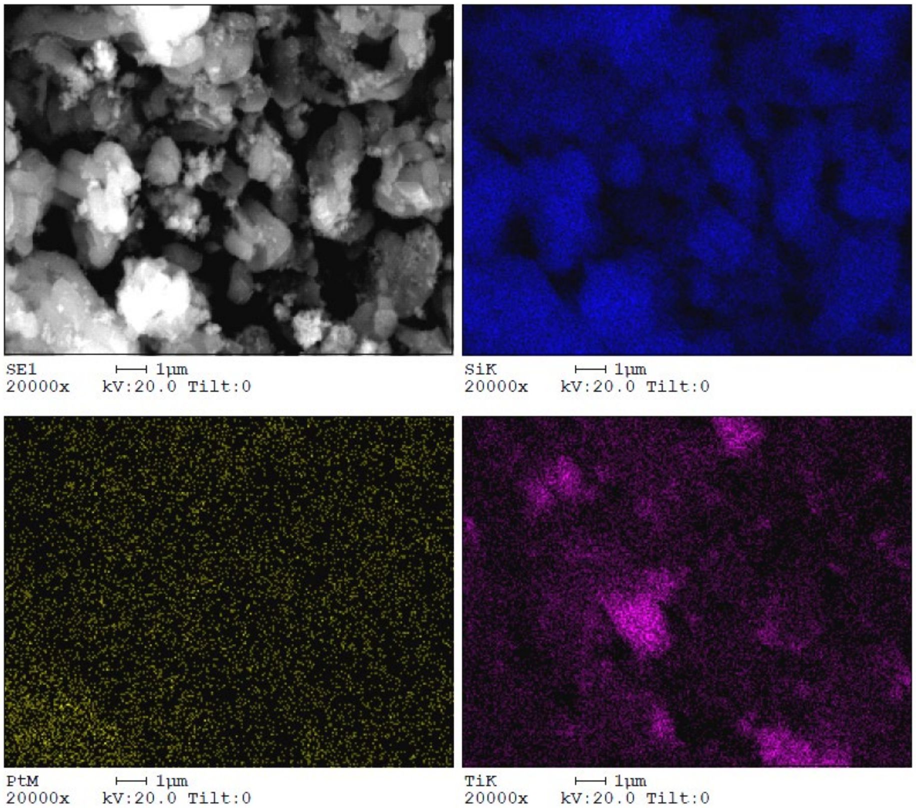

PT10SP

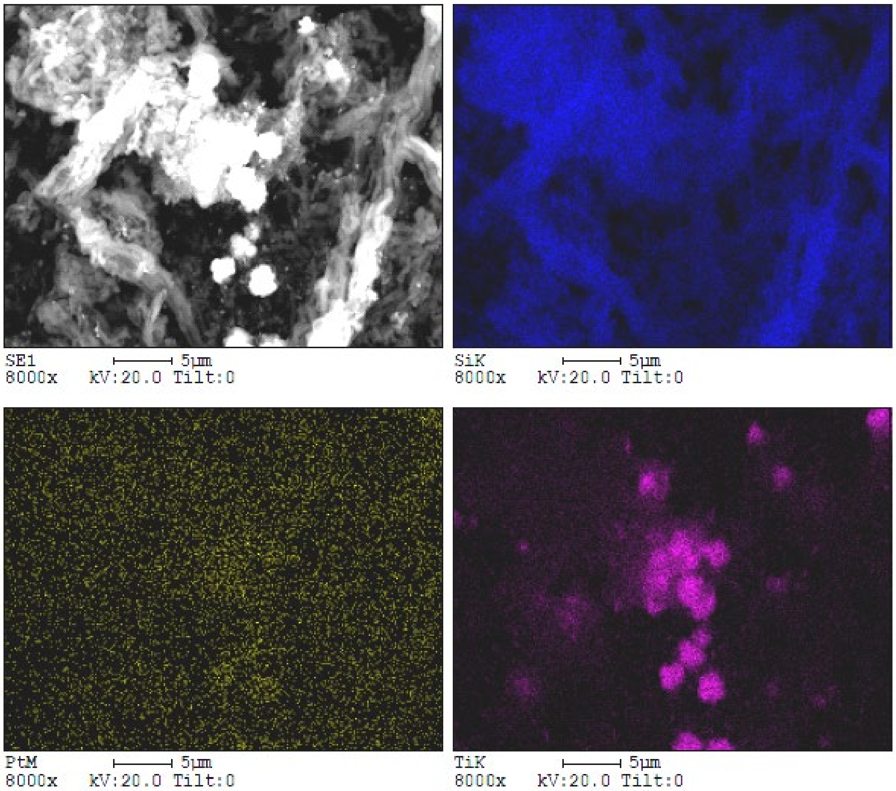

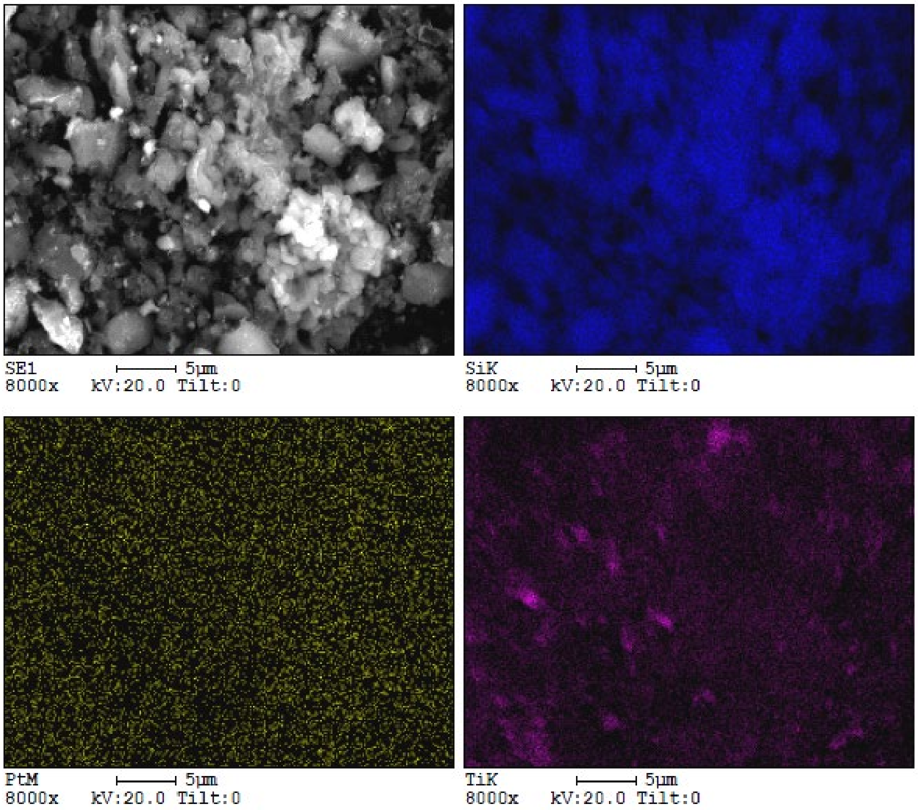

PT30SB

Figure S3. Elemental mapping images obtained by SEM.

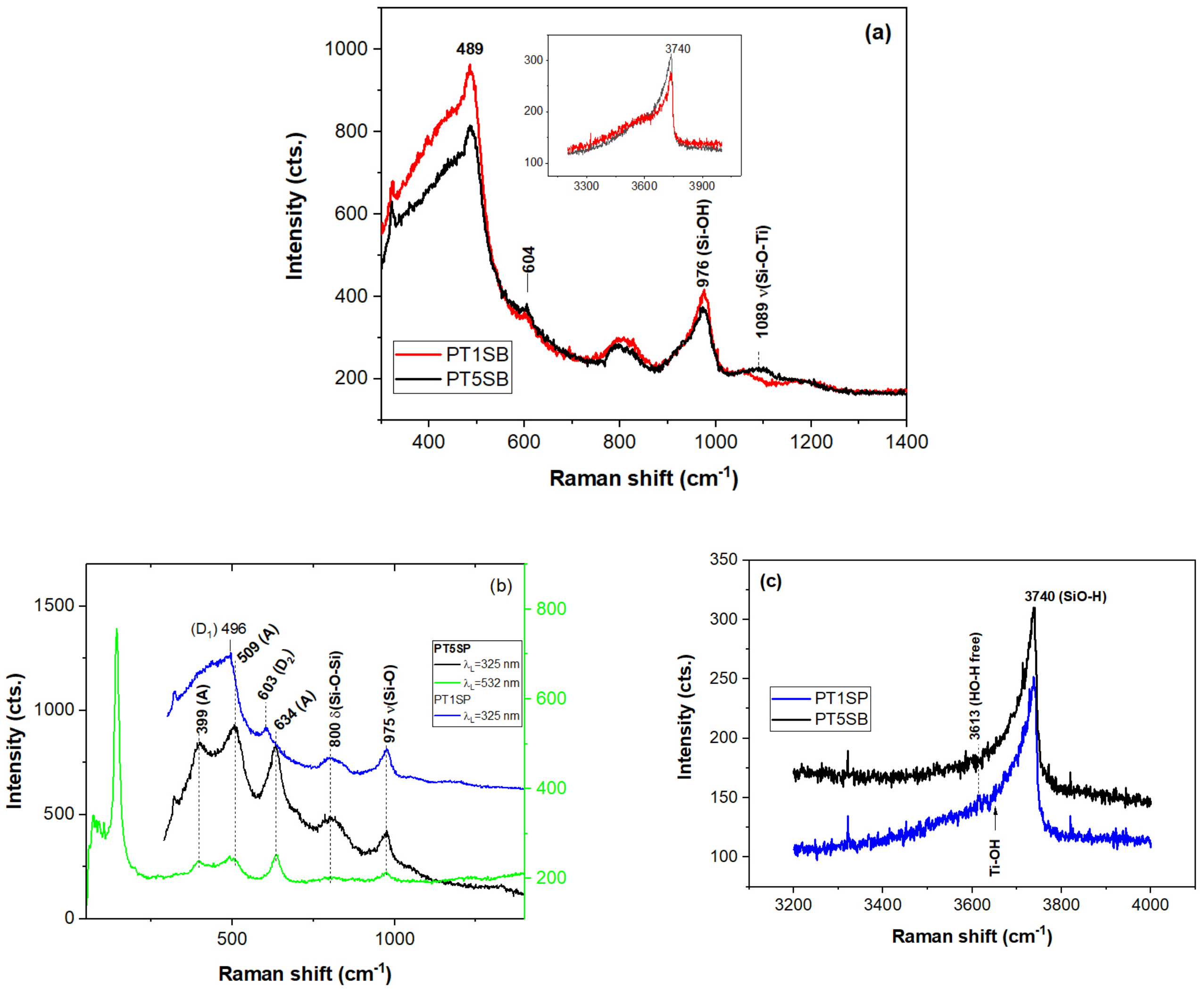

Figure S4. UV-Raman spectra of the PT(1/5)SB (a) and PT(1/5)SP (b,c) (A and R stand for anatase and Rutile). The Vis-Raman spectrum of the PT5SP was used for comparison.

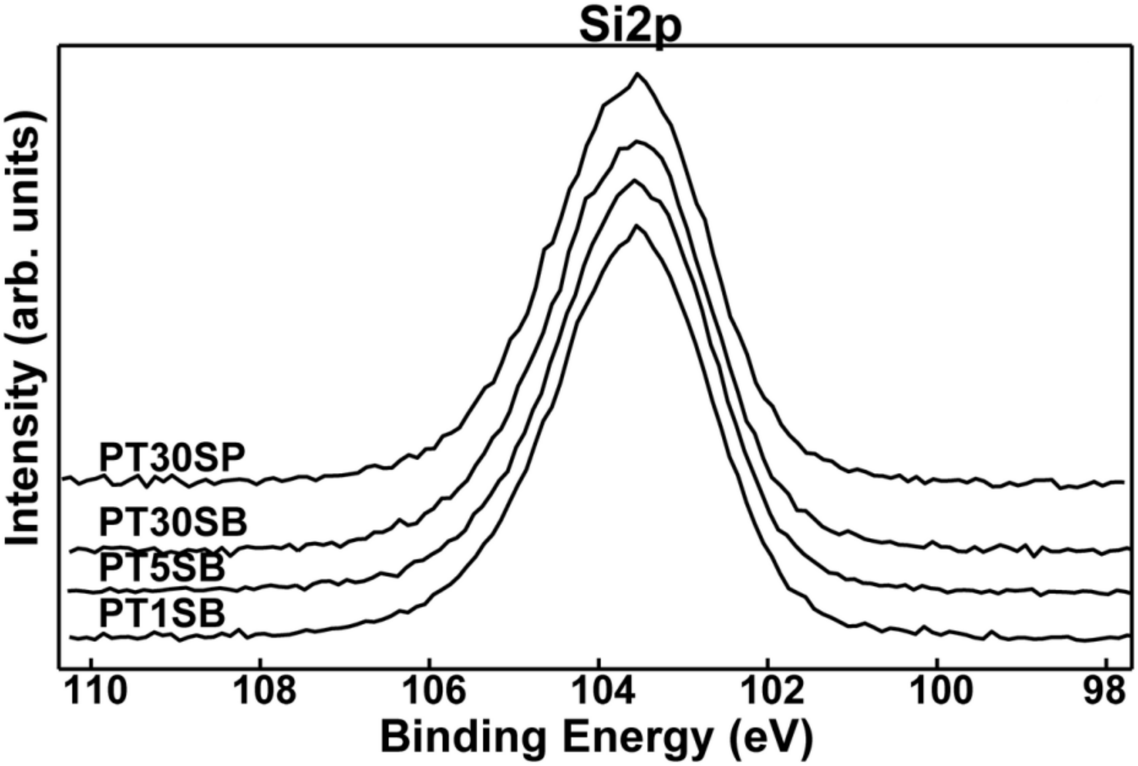

Figure S5. The XPS photoelectron spectra of the Si2p, superimposed spectra for PT1SB, PT5SB, PT30SB and PT30SP.
